# A new locus for autosomal dominant congenital coronary cataract in a Chinese family maps to chromosome 3q

**Published:** 2010-05-19

**Authors:** Guishun Liu, Yunbo Li, Yanfei Ruan, Wenping Cao, Li Xin, Jiangyuan Qian, Jingzhi Gu

**Affiliations:** 1 Department of Ophthalmology, the Port Hospital of Tianjin, Tianjin, China; 2 The First Hospital of Jilin University, Changchun, China; 3 Department of Ophthalmology, the First Affiliated Hospital of Harbin Medicine University, Harbin, China; 4 CAS Key Laboratory of Genome Sciences and Information, Beijing Institute of Genomics, Chinese Academy of Sciences, Beijing, China; 5 Department of Ophthalmology, the Second Affiliated Hospital of Harbin Medicine University, Harbin, China

## Abstract

**Purpose:**

To identify the genetic defect in an autosomal dominant congenital coronary cataract family (ADCCC).

**Methods:**

A Chinese family with ADCC was identified and characterized. All the members were genotyped with microsatellite markers at genes and loci that were considered to be associated with hereditary cataracts. Linkage analysis was performed after genotyping. Two-point Logarithm of odds (LOD) scores were calculated using MLINK software, from the LINKAGE program package. Multipoint parametric and non-parametric linkage were performed via the program MERLIN.

**Results:**

Linkage analysis provided evidence for a genetic locus for the ADCC on chromosome 3q. The maximum Two-point LOD score was 3.01 (θ=0) for two close markers.

**Conclusions:**

The mapping of the congenital cataracts in a Chinese family locus to chromosome 3q.

## Introduction

Congenital cataract is one of the significant causes of visual impairment and blindness in childhood, and it refers to opacification of the crystalline lens in infants. At least one-third of these cases are estimated to be familial [1]; most of them show an autosomal dominant pattern [2]. Congenital cataract may occur as an isolated, nonsyndromic form or as a component of a multisystem syndrome.

To date, more than 30 loci have been mapped for hereditary cataracts through linkage analysis on different chromosomes. Twenty genes have been identified to cause autosomal dominant congenital cataract (ADCC) including crystallin, alpha A (*CRYAA*) [3]; crystallin, alpha B (*CRYAB*) [4]; crystallin, beta A1 (*CRYBA1*) [5]; crystallin, beta B1 (*CRYBB1*) [6]; crystallin, beta B2 (*CRYBB2*) [7]; crystallin, gamma C (*CRYGC*) [8]; crystallin, gamma D (*CRYGD*) [9]; crystallin, gamma S (*CRYGS*) [10]; crystallin, beta A4 (*CRYBA4*) [11]; major intrinsic protein of lens fiber (*MIP*) [12]; gap junction protein, alpha 3 (*GJA3*) [13]; gap junction protein, alpha 8 (*GJA8*) [14]; beaded filament structural protein 2, phakinin (*BFSP2*) [15]; heat shock transcription factor 4 (*HSF4*) [16]; v-maf musculoaponeurotic fibrosarcoma oncogene homolog (avian; *MAF*) [17]; paired-like homeodomain 3 (*PITX3*) [18]; eyes absent homolog 1 (*EYA1*) [19]; forkhead box E3 (*FOXE3*) [20]; chromatin modifying protein 4B (*CHMP4B*) [21]; and ferritin, light polypeptide (*FTL*) [22]. Moreover, various ADCC-associated loci or chromosomal regions have been mapped where more genes are yet to be discovered. These include 1p36, 1q21-q25, 1q25-q31, 2p12, 2p24-pter, 2q33-q35, 3q21.2-q22.3, 13cen-q13, 14q22–23, 15q21–22, 16q22, 17p13, 17q11–12, 17q24, 19q, 20p12-q12, 21q22.3, and 22q11.2 [23,24].

Cataracts are clinically and genetically heterogeneous, since the same phenotype can be caused by more than one gene. On the other hand, different phenotypes can map to the same locus. Clinically, cataracts can be classified the following phenotypes: anterior polar, posterior polar, nuclear, lamellar (zonular), pulverulent, aculeiform, cerulean, cortical, polymorphic, sutural, and total cataracts [25]. Coronary cataract is an unusual type that has been rarely reported.

To identify the genetic defect in this family, we used allele sharing and linkage analysis methods in our study. The gene for congenital coronary cataract in this Chinese family was determined to be linked to chromosome 3q22.3-q25.2.

## Methods

### Family description and DNA isolation

**Figure 1:**
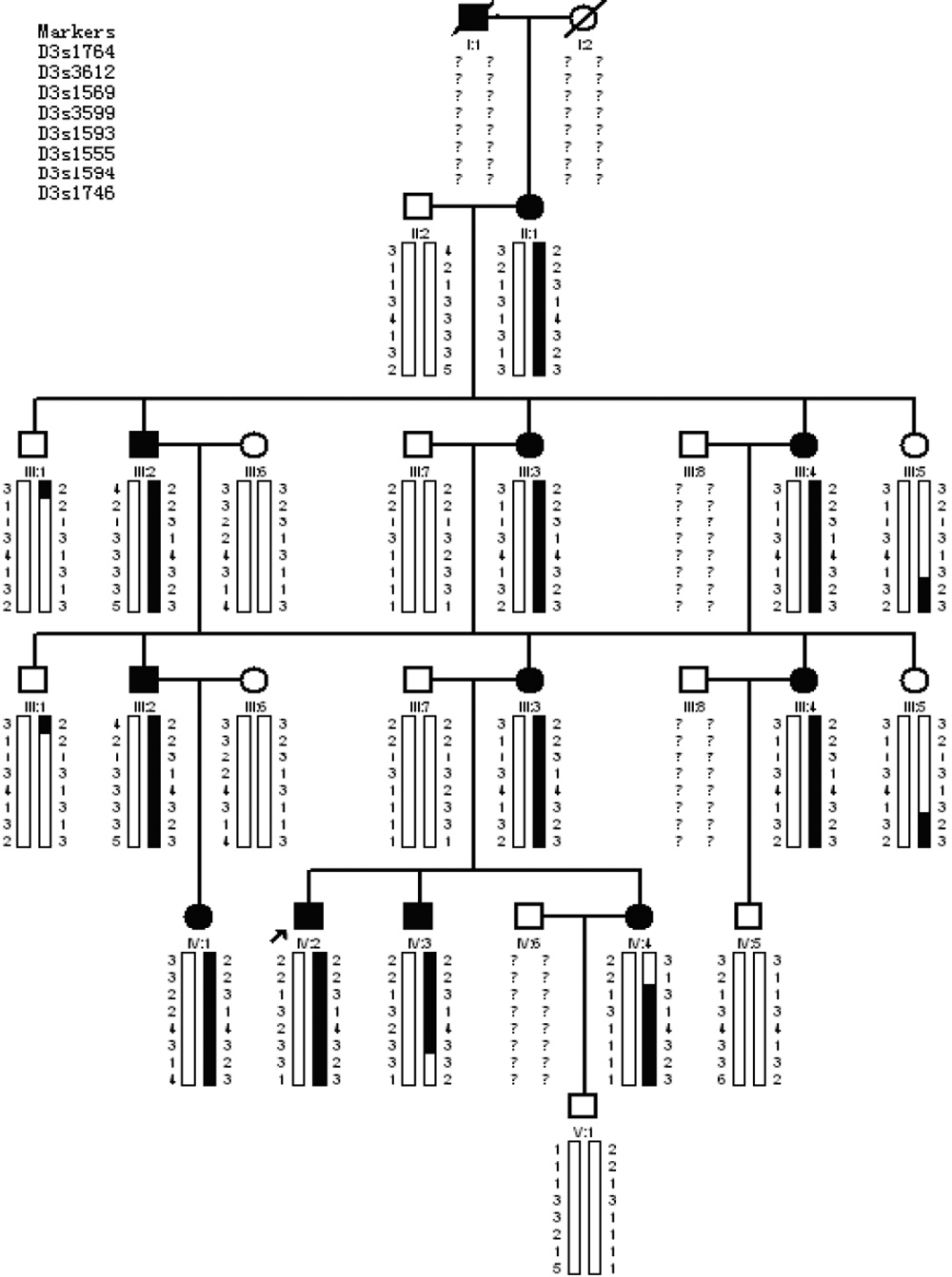
Pedigree and haplotype of the family with autosomal dominant congenital coronary cataract. The affected haplotype was indicated by a dark vertical bar, the normal haplotye by a white vertical bar.

A five-generation Chinese family with non-syndromic autosomal dominant congenital coronary cataract was investigated at the Department of Ophthalmology, the First Affiliated Hospital of Harbin Medicine University, Harbin, China. Fifteen family members, eight affected and seven unaffected, participated in this study and underwent a full ophthalmological examination (Figure 1).

The cataract was bilateral in all cases demonstrating club-shaped and focal dots opacities that distributed radially in the periphery of the cortex near the lens equator. The degree of the opacity increased with age in the affected individuals. None of the unaffected relatives had any evidence of cataracts. Venous blood (5 ml) was collected from each of the fifteen family members after informed consent from every family member. Briefly, Genomic DNA for subsequent molecular genetic analysis was extracted from peripheral blood leukocytes using a TIANamp DNA Blood Mini Kit (Tiangen Ltd., Beijing, China).

### Genotyping

We performed a partial genome scan around the 20 known causative genes and loci reported to be associated with ADCC. One hundred flanking microsatellite markers were chosen from the Marshfield genetic map with two markers for each known locus at least. Polymerase chain reactions (PCRs) were performed using Gene Pro (48/96/384/G; Bioer Ltd, Hangzhou, China) for these microsatellite markers. The primer sequences were obtained from NCBI and are presented in Table 1.

**Table 1:** Microsatellite markers for the known 20 loci reported to be associated with ADCC.

**Gene**	**Marker**	**Primer (F,R)**	**PCR product size (bp)**
*FOXE3*	D1S2720	F: AGCTACAAAGTGCTTTACTGACA	235-245
		R: AATGGTCCAGGCAAAGT	
	D1S232	F: GAGCAAGACTCTCTGTCCCC	184-202
		R: CCATGTTCAAGGGTCAACTG	
*GJA8*	D1S498	F: TTGCTGAAGGGACATAGTG	183-205
		R:TGCTGGGTTATATCCAATATC	
	D1S507	F: AGGGGATCTTGGCACTTGG	183-203
		R:CTCTAGGGTTTCTGGAAAATGCTG	
*CRYGC*	D2S1782	F: CTGACTTCACTGCTGTAATTGC	119-135
*CRYGD*		R: AAAGGCAAAGAAAGGGACTG	
	D2S2208	F: CTATTTGTAAACATGCGGGA	164-196
		R: AGCTAAGTACCTGCTCAGGAAA	
*CRYGS*	D3S1746	F: TCCCCTTTGAAAGTCCTACC	225-253
		R: TCCCTGGGTGAAAATGAGTA	
	D3S1594	F: CTGGNCACAGAGGGTC	266-334
		R: GCCACTTTTGCAGAGAACA	
*BFSP2*	D3S3612	F: TCCTTTGTTAGGCTCAATTTT	176-196
		R: TGCCCAGTTTTAGTCCA	
	D3S1764	F: TCCCCTTTGAAAGTCCTACC	225-253
		R: TCCCTGGGTGAAAATGAGTA	
*GCNT2*	D6S1717	F: GGGATGTTGAGAATGTTGTC	110-124
		R: GGGGGCTCTATCTCTAAAATT	
	D6S304	F: TTCCACTCTGCTCCAGACAG	228-250
		R: TTGAAAGTTTGAGAAGCACTTGTTA	
*EYA1*	D8S271	F: AGATGACCTGGATGAGAGTG	256-271
		R: AACAAACTTGCTTATGAGTGTTACT	
	D8S1840	F: TCCAGCCTGCATGACAGAGC	224-240
		R: TGTAACACTCACCGCGAGGG	
*PITX3*	D10S192	F: TTATACTAGGAAACAAGGCTTAC	179-198
		R: GGGCTTAAATGAATGAGCAC	
	D10S1760	F: GCGAGACTCCATCTCCATAG	112-155
		R: CCATATAGTGGGTGGCTTAAA	
*PAX6*	D11S904	F: ATGACAAGCAATCCTTGAGC	185-201
		R: CTGTGTTATATCCCTAAAGTGGTGA	
	D11S935	F: TACTAACCAAAAGAGTTGGGG	196-208
		R: CTATCATTCAGAAAATGTTGGC	
*CRYAB*	D11S2000	F: AGTAGAGAACAAAACACTGTGGC	199-235
		R: TTTGAAGATCTGTGAAATGTGC	
	D11S1998	F: AGCCATCAACTAGCTTTCCC	129-165
		R: GGGAGGCACCAACAGATG	
*MIP*	D12S1632	F: GCCTAATCAAGATGTCACCA	216-226
		R: GCTAGGGAGCCAATTCA	
	D12S1691	F: GGTAAACACTGAGACACGCC	194-230
		R: TGATGACNCAGAAGTTGAGC	
*GJA3*	D13S1275	F: ATCACTTGAATAAGAAGCCATTTG	180-214
		R: CCAGCATGACCTTTACCAG	
	D13S175	F: TATTGGATACTTGAATCTGCTG	101-113
		R: TGCATCACCTCACATAGGTTA	
*CHX10*	D14S71	F: TGCACCAATGCCTCCT	191-211
		R: CCCGGCCAGAAATGCT	
	D14S81	F: CAGAGAAATGAGTTGAGTATGGTT	175-209
		R: CAACAGAGCAGGACCCTTTC	
*HSF4*	D16S3067	F: GCCACCTCACACTAGCCTG	138-152
		R: TCACTCAAAATGGAGTCACTCTG	
	D16S421	F: ACATGAACCGATTGGACTGA	206-216
		R: CCGTTCCCTATATTTCCTGG	
*MAF*	D16S3101	F: TTCCTGAATGTCATGTAGTTGG	158-166
		R: TGTCATCGGGGCTTGTAG	
	D16S3119	F: TAGGCATGACTGGGGGT	293-305
		R: TGAACTAACACAAGCAGCCA	
*CRYBA1*	D17S1294	F: TGGCATGCAATTGTAGTCTC	248-272
		R: TTCTTTCCTTACTAAGTTGAGAACG	
	D17S1857	F: TGCACAGGCCAATTCCTTAC	177-187
		R: TGCCTAAACTGCTTTCAGGTGAG	
*FTL*	D19S902	F: CCATCCTAATGAGGGCAA	199-217
		R: GCACCAGTGACTGCCTGT	
	D19S585	F: AGCACCATAATGCCTGGATA	103-127
		R: CAGCATAGCAAGACCCTGTC	
*CHMP4B*	D20S195	F: GTACCTCCTCCAGGCTTC	237-261
		R: AGGGGTGTATGTGTGCAT	
	D20S847	F: AAGCCGGAAATCTGGACTT	113-145
		R: ATGTGGTTTGGGTTCCCT	
*CRYAA*	D21S1890	F: GGTCTGACCACAGATTTCC	143-173
		R: AAAAACACTCTGAACGATTAAGG	
	D21S1260	F: TCCAAGGGGTTCATCC	200-214
		R: CCCAAGGCACTGTTCC	
*CRYBB1*	D22S1167	F: ACATGGCAAAACCCAGTCTC	266-278
*CRYBB2*		R: GGGGCTTCAACAACATTCTTAAC	
*CRYBA4*	D22S419	F: GGCTCAGGGACTCTGGA	257-273
		R: GGCCAATCGGTAGGTCA	
	D22S351	F: GTCAGGGCAGGTAAGGTTGA	145-149
		R: CTCCTGCCCTCGAAAGTCAT	

In the Table, F indicates Forward primer and R indicates Reverse primer.

Microsatellites were amplified in a 25 μl reaction volume. The conditions of PCR are as follows: denaturation at 95 °C for 5 min followed by 35 cycles of denaturation at 95 °C for 30 s, annealing at 54–60 °C for 30–35s, and extension at 72 °C for 30 s with the last extension for 8 min at 72 °C. PCR products from each DNA sample (2–4 μl) were mixed with denaturants (0.05% bromophenol blue, 0.05% Xylene Cyanol FF, 1 mmol/l EDTA, and 95% deionized formamide; Solarbio Ltd, Biejing, China) in equal volume and were denatured at 95 °C for 5 min, pooled, and were separated on 8% denaturing polyacrylamide gels. Cyrillic (version 2.1) software was used to manage the pedigree and haplotype data. We performed exclusion analysis by allele sharing in affected family members.

### Linkage analysis

Two-point Logarithm of odds (LOD) scores were calculated by MLINK of the LINKAGE package (version 5.1). A gene frequency of 0.0001 and a penetrance of 0.9999 were modeled for ADCC. The allele frequencies for all markers were considered to be equally distributed in males and females. Multipoint parametric and non-parametric linkage was performed via the program MERLIN, version 1.01.

## Results

### Exclusion analysis

First, the 20 known candidate genes for ADCC were excluded on human chromosome 1q, 1p, 2q (two genes), 3q (two genes), 8q, 10q, 11q, 12q, 13q, 16q (two genes), 17q, 19q, 20q, 21q, and 22q (three genes). Second, a linkage analysis of the other 17 known loci was performed, including chromosome 1p36, 1q21-q25, 1q25-q31, 2p12, 2p24-pter, 2q33-q35, 3q21.2-q22.3, 13cen-q13, 14q22–23, 15q21–22, 17p13, 17q11–12, 17q24, 19q, 20p12-q12, 21q22.3, and 22q11.2. Recombinant individuals were observed and all LOD scores were <-2 in the excluded known loci (data not shown).

### Linkage analysis

While attempting to exclude linkage with beaded filament structural protein 2, phakinin (*BFSP2*) and crystallin, gamma S (*CRYGS*) on chromosome 3q, we obtained significant positive LOD scores for marker D3S1569 (*Zmax*=3.01, θ=0.00). Two-point analysis succeeded to confirm evidence of linkage and gave a maximum LOD score of 3.01 at θ=0.00 with D3S1569 and D3S3599 (Table 2). Results of multipoint analysis are presented in Table 3. The maximum multipoint LOD score (3.01) occurs across the 16.98-cM region from D3S1764 to D3S1746.The co-segregating region lies in the 15.2-cM interval from D3S3612 to D3S1594 by haplotype analysis (Figure 1). This study provides the first genetic mapping of an autosomal dominant congenital coronary cataract that corresponds to the q22.3–25.2 region of chromosome 3. The candidate genes, *BFSP2* and *CRYGS* on chromosome 3q, are far outside of the critical interval.

**Table 2:** Two-point LOD scores for microsatellite markers on chromosome 3q22.3–25.2 in the Chinese ADCC family.

		**LOD score at a recombination fraction of θ**					
**Marker**	**Genetic position (cM)**	**0.0**	**0.1**	**0.2**	**0.3**	**0.4**	**0.5**
D3S1764	152.62	–∞	0.60	0.75	0.60	0.33	0.00
D3S3612	153.74	–∞	–0.19	0.01	0.07	0.06	0.00
D3S1569	158.38	3.01	2.51	1.94	1.31	0.62	0.00
D3S3599	158.38	3.01	2.51	1.94	1.31	0.62	0.00
D3S1593	161.04	1.81	1.49	1.13	0.73	0.31	0.00
D3S1555	165.32	0.90	0.73	0.54	0.36	0.18	0.00
D3S1594	168.94	–∞	0.60	0.75	0.60	0.33	0.00
D3S1746	169.60	–∞	0.58	0.62	0.51	0.30	0.00

**Table 3:** Multipoint linkage analysis spanning 16.98 cM interval from D3S1764 to D3S1746 on chromosome 3 using the program MERLIN version 1.01.

**Marker**	**Position (cM)**	**LOD score (PL)**	**LOD score (NPL)**	**p-value**
D3S1764	152.62	−12.99	1.06	0.14
D3S3612	153.74	−8.049	1.05	0.15
D3S1569	158.38	3.01	5.55	0
D3S3599	158.38	3.01	5.55	0
D3S1593	161.04	3.009	5.53	0
D3S1555	165.32	2.689	5.51	0
D3S1594	168.94	−8.99	1.1	0.14
D3S1746	169.60	−7.379	1.08	0.14

In the Table, PL indicates parametric linkage and NPL indicates non-parametric linkage.

## Discussion

In this study, a locus on chromosome 3 in an ADCC family of Chinese origin having coronary cataract was identified. The phenotype takes on shapes of club-shaped, oval, and discrete opacities. The opacities of the cataract in this family occur in only one region of the lens, the deep cortex and distribute in a radial pattern surrounding the nucleus like a crown, so the affected individuals are usually asymptomatic,that is to say there is no influence on the patients’ eyesight. Furthermore, no other systemic or ocular disorders have been found in the affected individuals. Coronary cataract is an unusual phenotype and is seldom reported in genetic research [26]. By now, considerable phenotypic variability occurred between the locus in our family and the other loci reported on chromosome 3 [24,27-29].

Sidjanin et al. [30] mapped a mouse autosomal dominant cataract mutation *(Coc)* to the region of Dl6Mit12 and D16Mit38, which was ~26 cM distal to the centromere on mouse chromosome 16 and in a syntenic region with human chromosome 3q21-q24. Therefore, the *Coc* locus may be homologous to the human cataract locus in our present study.

In conclusion, a new locus for ADCC has been identified on human chromosome 3q22.3–25.2. Affected individuals of this Chinese family exhibit an unusual coronary phenotype. Further study is needed to find candidate genes and give insights into the molecular mechanisms of the cataract formation in this family. And it maybe provide molecular evidence for clinical differential diagnosis.
